# A Text Messaging Intervention to Support Latinx Family Caregivers of Individuals With Dementia (CuidaTEXT): Development and Usability Study

**DOI:** 10.2196/35625

**Published:** 2022-04-28

**Authors:** Jaime Perales-Puchalt, Mariola Acosta-Rullán, Mariana Ramírez-Mantilla, Paul Espinoza-Kissell, Eric Vidoni, Michelle Niedens, Edward Ellerbeck, Ladson Hinton, Linda Loera, A Susana Ramírez, Esther Lara, Amber Watts, Kristine Williams, Jason Resendez, Jeffrey Burns

**Affiliations:** 1 Alzheimer’s Disease Research Center School of Medicine University of Kansas Medical Center Fairway, KS United States; 2 Department of Population Health School of Medicine University of Kansas Medical Center Kansas City, KS United States; 3 Alzheimer's Disease Research Center University of California, Davis Sacramento, CA United States; 4 Alzheimer’s Association, Southland Chapter Los Angeles, CA United States; 5 Department of Public Health University of California, Merced Merced, CA United States; 6 Alzheimer’s Disease Research Center Department of Psychology University of Kansas Lawrence, KS United States; 7 Alzheimer’s Disease Research Center School of Nursing University of Kansas Medical Center Fairway, KS United States; 8 UsAgainstAlzheimer’s Washington, DC United States

**Keywords:** Latinx individuals, mHealth, dementia, caregiving

## Abstract

**Background:**

Latinx family caregivers of individuals with dementia face many barriers to caregiver support access. Interventions to alleviate these barriers are urgently needed.

**Objective:**

This study aimed to describe the development of *CuidaTEXT*, a tailored SMS text messaging intervention to support Latinx family caregivers of individuals with dementia.

**Methods:**

*CuidaTEXT* is informed by the stress process framework and social cognitive theory. We developed and refined *CuidaTEXT* using a mixed methods approach that included thematic analysis and descriptive statistics. We followed 6 user-centered design stages, namely, the selection of design principles, software vendor collaboration, evidence-based foundation, caregiver and research and clinical advisory board guidance, sketching and prototyping, and usability testing of the prototype of *CuidaTEXT* among 5 Latinx caregivers.

**Results:**

*CuidaTEXT* is a bilingual 6-month-long SMS text messaging–based intervention tailored to caregiver needs that includes 1-3 daily automatic messages (n=244) about logistics, dementia education, self-care, social support, end of life, care of the person with dementia, behavioral symptoms, and problem-solving strategies; 783 keyword-driven text messages for further help with the aforementioned topics; live chat interaction with a coach for further help; and a 19-page reference booklet summarizing the purpose and functions of the intervention. The 5 Latinx caregivers who used the prototype of *CuidaTEXT* scored an average of 97 out of 100 on the System Usability Scale.

**Conclusions:**

*CuidaTEXT*’s prototype demonstrated high usability among Latinx caregivers. *CuidaTEXT*’s feasibility is ready to be tested.

## Introduction

### Background

Family caregiving for individuals with dementia has a serious emotional, physical, and financial toll [1-8]. Most individuals with dementia live at home and are cared for by their relatives [9]. As the US health care system focuses mainly on acute care, relatives provide >80% of the long-term care for individuals with dementia [4,5]. For these reasons, caregiver support is a key component of the National Alzheimer’s Project Act [10].

Most family caregiver interventions have been designed for non-Latinx White individuals, and the results might not generalize to other groups because of linguistic, cultural, and contextual reasons [11-13]. The number of Latinx individuals with dementia is projected to increase from 379,000 in 2012 to 3.5 million by 2060, more than any other group [14]. Latinx individuals are more likely to become family caregivers than non-Latinx White individuals [15]. Latinx individuals also provide more intense and longer caregiving and experience higher levels of caregiver depression and burden [8,15-21]. However, despite their high interest in participating in caregiver support interventions [22], Latinx caregivers of individuals with dementia are less likely to use caregiver support services [23,24]. This disparity is partly due to Latinx caregivers’ more frequently experienced barriers related to transportation, financial, language, and cultural aspects compared with non-Latinx White caregivers [23,24]. Therefore, the need for targeted caregiver support interventions among Latinx individuals is crucial. This need is in line with the National Institute on Aging’s call to address health disparities in aging research [25].

SMS text messaging offers distinct advantages over websites and apps for delivering interventions [26-28]. Although nearly all Latinx individuals engage in SMS text messaging, Latinx individuals’ low use of websites and apps could perpetuate disparities in access to caregiving support [29]. Caregiver interventions for Latinx individuals need to capitalize on SMS text messaging, as SMS text messaging interventions (1) are effective in treating or preventing other health conditions such as tobacco addiction or diabetes; (2) can be used anywhere at any time; (3) are more cost-effective than other delivery systems; (4) can be personalized to caregivers’ preferences and characteristics including language, culture, and needs; (5) are highly scalable among Latinx individuals, as most own a cell phone with SMS texting capabilities, more than other groups; and (6) have been specifically shown to engage Latinx individuals [30-35].

### Objectives

To address Latinx individuals’ disparities in access to caregiving support, we developed *CuidaTEXT* (a Spanish play on words for self-care and texting). To our knowledge, this is the first SMS text messaging intervention for caregiver support of individuals with dementia among Latinx individuals or any other ethnic group. Only one other SMS text messaging intervention exists in the context of dementia [36]. However, that intervention was designed to increase dementia literacy among non-Latinx Black users and is not geared toward Latinx individuals or caregivers specifically. The aim of this study was to describe the development of *CuidaTEXT*, a tailored SMS text messaging intervention to support Latinx family caregivers of individuals with dementia. This development corresponds to Stage 1a of the National Institutes of Health Stage Model for Behavioral Intervention Development (intervention generation) [37]. This intervention will later be feasibility-tested (Stage 1b) among Latinx family caregivers of 20 individuals with dementia (ClinicalTrials.gov NCT04316104).

## Methods

### Overview

This was a mixed methods project guided by user-centered design principles [38]. The basis for user-centered design is that gathering and incorporating feedback from users into product design will lead to a more usable and acceptable product. Mixed methods are required, given the lack of literature on SMS text messaging interventions for Latinx family caregivers of individuals with dementia and the strengths of a combined qualitative and quantitative approach [39]. We followed 6 user-centered design stages informed by previous research used to develop successful behavioral intervention software [40]. The user-centered design stages are described in the next sections.

### Ethics Approval

All study procedures were approved by the institutional review board of the University of Kansas Medical Center (STUDY00144478). All participants provided written informed consent.

### Stage 1: Selection of Design Principles

A total of 2 design principles were specified. First, we selected the social cognitive theory as the main behavior change principle [41]. This principle has been successfully used in previous SMS text messaging interventions [30]. The social cognitive theory informs the identification of barriers to desired behaviors, setting of realistic goals, encouragement of gradual practice to achieve performance accomplishments of healthy behaviors (eg, relaxation techniques or exercising), integration of testimonials and videos to promote vicarious learning, integration of praise to elicit social persuasion, and education to increase dementia knowledge. Second, we chose the stress process framework [42] to guide the development of messages to encourage coping and social support behaviors (mediators), which are aimed at improving role strains (eg, perceived income adequacy and family interaction), intrapsychic strains (eg, mastery, self-esteem, and loss of self), and, ultimately, outcomes (eg, caregiver depression, affect, and self-perceived health).

### Stage 2: Vendor Collaboration for Text Messaging Design and Delivery

This stage aimed to materialize the vision and design specifications of *CuidaTEXT*. We developed a checklist of necessary features to identify potential vendors, including message personalization, 2-way SMS text messaging, scheduling, conditional branching logic for SMS text message responses, information tracking, technical support, and cost. We identified 3 vendors based on our previous experiences and a basic internet search. The 3 identified vendors met all the features, and we selected the one with the most affordable cost. Their services also included configuration, account setup, initial onboarding, training guides and videos, access to the vendor system, mobile number support, and bug fixes during the intervention. We contracted with them early in the project to avoid delays (eg, developing the scope of work, registering as a vendor, contracts, and software programming).

### Stage 3: Evidence-Based Foundation

This stage aimed to identify core content categories based on previous successful behavioral interventions. We searched specifically for general caregiver support interventions considered evidence-based informed by the Administration for Community Living [43], PubMed literature results using the Medical Subject Headings terms *Caregivers*, *Dementia*, and *Hispanic Americans*, and recommendations on behavioral interventions from the research team. Content categories included dementia education, problem-solving skills training, social network support, care management, and referral to community resources [1,26,27,44-58].

### Stage 4: Advisory Board Guidance

Advisory board guidance provided expert opinion to inform the SMS text messaging intervention based on Latinx caregiver needs [22,40,59,60]. We conducted 5 parallel advisory board meetings with up to 6 Latinx caregivers and 16 clinicians and researchers (health professionals), each lasting 60 minutes. We used purposive sampling for the Latinx caregivers and quota sampling for the health professionals (including at least one person with expertise in psychiatry, social work, neurology, dementia care interventions, Latinx research, SMS text messaging intervention development, or behavioral health). We conducted the caregiver advisory board sessions in Spanish and the health professional group sessions in English. We held all sessions via videoconference from December 2020 to May 2021 and recorded each to facilitate notetaking and analysis. The process for each meeting was similar: the research team showed the groups a step-by-step explanation of the components of the study, asked specific questions pertinent to the phase of the study, and then facilitated open discussion about the project. The research team took detailed notes of all sessions, which were used for further analysis. We organized the notes for qualitative review using a pragmatic approach, a qualitative description methodology, and thematic analysis methods [61-63]. We coded the content of the notes using Microsoft Word by identifying codes and themes within the text [64]. In addition, 2 researchers (JPP and MAR) independently reviewed the codes and resolved coding disagreements through discussion and consensus.

### Stage 5: Sketching and Prototyping

On the basis of the previous stages, 3 researchers (JPP, MAR, and PEK) brainstormed a pool of potential SMS text messages in English on a shared spreadsheet and later sorted the messages by topic (initial draft keywords). We edited messages following the *Seven Principles of Communication*: completeness, concreteness, courtesy, correctness, clarity, consideration, and conciseness [65]. This theory is popular in business communications and has been used in patient reporting [66,67]. Bilingual, bicultural members of the research team translated the messages into the primary Spanish dialects represented in the United States (Mexican and Caribbean).

In addition to the SMS text message libraries, we developed a reference booklet for participants that summarized the purpose of the intervention and its functions. The booklet is not necessary to use *CuidaTEXT*. However, the research team considered it to be useful for those who want to learn about the intervention faster or increase personal sense of agency. On the basis of our previous development experience with the Latinx community [22,30], we made the booklet available in both English and Spanish and used lay language and a pictorial format. A total of 7 research team members tested the SMS text messaging prototype powered by the vendor on their own cell phones from early June to August 2021 and provided feedback that was used for message refinement iteratively, as suggested by the literature [68,69]. We recorded the feedback via SMS text message responses within the vendor platform and emails from the research team to the vendor’s programmer. We organized the data (SMS text messages and emails) for qualitative review using a process identical to that described in Stage 4.

### Stage 6: Usability Testing

Usability testing aimed to test a short prototype of the SMS text messaging intervention and assessments among actual Latinx caregivers. We used the vendor’s platform to preview the behavior and opinions of diverse Latinx caregivers in a variety of key scenarios (ie, reading specific messages, using keywords, sending SMS text messages, opening links to websites and videos, and downloading PDF files). The testing sessions were conducted via videoconference in June 2021, lasted approximately 90 minutes per person, and were conducted in English and Spanish based on participant preferences. We took detailed notes of the observations during the usability testing sessions and participants’ comments at the end.

### Sample and Assessment

We recruited 5 individuals, as suggested by software development cost-benefit analyses [70]. In this framework, the first participant discovers most flaws, and after the fifth user, findings tend to repeat without learning much new. Participants were recruited from 3 previous projects at the research center using purposive sampling. Eligibility criteria included Spanish- or English-speaking individuals who were aged 18 years or older, identified as Latinx, reported providing care for a relative with a clinical or research dementia diagnosis, and with an Ascertain Dementia 8cognitive screening score ≥2, indicating cognitive impairment [71,72]. Participants also had to report owning a cell phone and being able to use it to read and send SMS text messages. Participants received US $20 prepaid gift cards for completing assessments. We used 3 usability evaluation modalities. First, direct observation of task completion (eg, texting keywords) with the intervention prototype via monitoring of participants’ SMS text message responses was conducted. Second, open-ended interviews of user experience with the different tasks and suggested changes to improve the intervention (see Multimedia Appendix 1 for a sample) were conducted. Third, the caregiver participants completed the System Usability Scale about their experience with the prototype [73]. The System Usability Scale is a valid and reliable 10-item, 5-point Likert scale. According to the developers of the scale, scores >68 out of 100 indicate higher levels of usability. We modified the design features iteratively after each participant and provided the new version to the following participant, as suggested by the literature [74]. After 2 consecutive participants reviewed and approved an SMS text message, the following participant received an SMS text message with different features. In addition to the usability evaluation, we administered a survey to gather baseline characteristics.

### Data Analysis

We analyzed the qualitative data (detailed notes) from the open-ended interviews using a process identical to that described in Stage 4. We analyzed quantitative baseline characteristics using central tendency estimates, frequencies, and percentages on SPSS software (IBM Corp) [75].

## Results

### Overview

This section focuses on Stages 4 to 6 of the user-centered design proposed in this project. First, we summarize the findings from each stage. Second, we explain how these findings informed the intervention development within each stage. Third, we describe the final version of the intervention.

### Findings From Advisory Board Guidance (Stage 4)

Table 1 shows the themes, subthemes, and descriptions of the aspects to be considered in the development of *CuidaTEXT*, according to the advisory boards. Both Latinx caregivers and health professionals contributed to all the themes. Building on the evidence-based foundation established in Stage 3, feedback from this stage informed the sketching and prototyping of *CuidaTEXT*. First, the advisory board emphasized that messages should include specific content on logistics (eg, guidance on *CuidaTEXT*’s functions and motivation messages), social support, caregiver needs, care recipient needs, preparation for the care recipient’s death, and reminders for physician visits or medicines. This feedback informed the addition of the suggested SMS text message content. Second, advisory board members highlighted the importance of allowing the inclusion of >1 relative within the family to reduce burden and increase social support. This feedback informed the decision to enroll >1 caregiver per individual with dementia in future studies. Third, the advisory board suggested that the domains of SMS text messages sent to caregivers should alternate often and be tailored to the needs of caregivers. This feedback informed the inclusion of high-priority message content at the beginning of the intervention (eg, who to contact in case of elder abuse or suicidal thoughts and removing weapons in the home) and the frequent alternation of domains. This feedback also reinforced the need to use 2-way SMS text messaging, as originally planned. Fourth, the preferences of caregiver advisory board members varied widely with respect to the number of messages per day *CuidaTEXT* should send participants. A participant emphasized that they would abandon the intervention if they received >1 message per day except at the beginning, which required more messaging. Others wanted to receive 5 or more messages per day. Eventually, a consensus was reached that *CuidaTEXT* should tailor the number of messages to the preferences of caregivers. This feedback informed the decision to send few daily automatic messages per day to participants (generally 1). Fifth, the advisory board emphasized the need to make keyword names as simple and recognizable as possible and suggested several edits in line with this idea. This feedback informed the refinement of some keyword names. Sixth, the advisory board suggested adding diverse information regarding COVID-19. However, after some discussion, a consensus was met not to develop automatic SMS text messages for *CuidaTEXT*, given the rapid evolution of COVID-19 information. This feedback informed the exclusion of COVID-19 automated information into *CuidaTEXT*. Seventh, an advisory board member, guided by her experience, highlighted the scarce existing resources for caregivers with hearing issues and suggested that *CuidaTEXT* was made as *hearing impairment friendly* as possible. This feedback reinforced the idea of delivering the intervention via SMS text messaging. This feedback also informed the use of hearing impairment–inclusive messaging, including SMS text messaging–based contact information of all shared resources (eg, text telephone contact numbers) and video links with closed caption subtitles. Eighth, the advisory board suggested several *CuidaTEXT* reference booklet edits for simplification. This feedback informed the refinement of the *CuidaTEXT* reference booklet.

**Table 1:** Themes, subthemes, and descriptions of topics elicited during the advisory board sessions with Latinx caregivers and health professionals.

Themes and subthemes		Description
**Messages should include specific content**		
	Logistics	Motivate participants to be in the program
	Dementia education	Address the whole family to be all on the same page; dementia stages and variation between individuals; signs and symptoms; address stigma by normalizing dementia
	Social support	Communication with the individual with dementia; include concrete examples (eg, allowing individuals with dementia enough time to answer) and resources (eg, Alzheimer’s Association, health professionals, services in Spanish, support groups, and legal assistance); communication with the health provider (eg, expectations, what to report, encouraging clinical diagnosis, and requesting interpreters); improve family communication (eg, understanding family roles and find strengths, knowing importance of family support, and disclosing the diagnosis to the family); and develop active listening skills (eg, reflecting and nonverbal language)
	Caregiver needs	Cope with depression, anxiety, and stress (eg, relaxation); choose one’s battles; use sense of humor; find positive aspects of caregiving; address loneliness; cope with loneliness; capitalize on spirituality; and maintain a healthy lifestyle
	Care recipient needs	Address behavioral symptoms as specifically as possible; address aggressive behavior (eg, understanding the disease causing it, distraction techniques, and communication when the person is aggressive); address anxious behavior (distraction techniques and prevention); help the individual with dementia; address daily care of the individual with dementia, including healthy eating, dressing, hygiene, and doing fulfilling activities
	Preparation for the care recipients’ death	Information about what to expect at the end of the life of the individual with dementia; grieving strategies and tips
	Appointments for the physician or medicine	Set notifications to remind caregivers of medications or physician visits
Need to integrate other family members		More than 1 family member should be allowed to participate, to share responsibilities, avoid burdening a caregiver by having to educate the rest, promote collective understanding about what the individual with dementia is experiencing, and reduce the isolation of the caregiving process
Messages should follow a certain order		Alternate content often (eg, educational, caregiver tips, and resources) and tailor content to caregivers’ needs
Messages should have a certain dose		Limit mandatory message frequency to 1 per day (in general) and tailor frequency and timing to caregivers’ preferences
How to integrate COVID-19 into *CuidaTEXT*		Reliable education about caring during COVID-19, including what to do if infected, risks, vaccines, and vaccine locations and resources for those experiencing technological divide. As changes in COVID-19 evolve quickly, it was decided not to automate messages and send only ad hoc information as needed
Some keyword names need editing		Use simple and recognizable keyword names if possible; allow platform recognition of alternative spellings and typos; and edit specific keywords: *STRESS* vs *RELAX*, *GRIEF* vs *ENDOFLIFE* or *LOSS*, *BANO* vs *ORINAR*, *DUELO* vs *FINDEVIDA*, *PACIENTE* vs *SERQUERIDO*
*CuidaTEXT* could benefit people with hearing impairment		Given that SMS text messages are visual, this intervention can be optimized for people with hearing impairment
Reference booklet needs editing		Shorten booklet and include fewer and more realistic examples by including a matrix of the different keyword messages

### Findings From Sketching and Prototyping (Stage 5)

Table 2 shows the themes, subthemes, and descriptions of aspects considered in the development of *CuidaTEXT* based on the feedback from SMS text messages from the team during the testing of the prototype and suggestions provided by the vendor’s programmer. These aspects led to several solutions. First, we allowed the platform to recognize common misspelling or alternative spellings for keywords (eg, for the keyword *Behavior*, the platform should also accept *Behaviour*, *Behaviors*, *Behaviours*, and *Behavour*). Second, we edited words to eliminate misspellings or replace words that required high literacy levels or specialized knowledge (eg, glutes vs rear). Third, we used a vendor-owned link shortener to save SMS text message characters, as using a third-party link shortener could lead to phone carriers identifying messages as spam and subsequently blocking them. Fourth, we added code to embed the participant’s first name in SMS text messages to personalize them. Fifth, we adjusted the time of delivery of each daily automatic SMS text message to account for participants’ time zone. Sixth, we requested that the vendor allow keyword libraries to loop back to the first SMS text message after reaching the last one on the list.

**Table 2:** Themes, subthemes, and descriptions of topics elicited during the sketching and prototyping stage, gathered via SMS text message responses from the team and correspondence with the SMS text messaging vendor.

Themes and subthemes		Description
**Testing actions**		
	Testing keyword responses	Ensuring the platform responded with an automatic SMS text message instantly upon sending a specific keyword
	Testing live chat	Ensuring the platform received SMS text messages other than keywords, intended for the coach, such as “thank you!”
	Testing alternative spellings of keywords	Ensuring alternative spellings and misspellings of keywords are recognized by the platform as such
	Testing links and PDF downloads	Ensuring links to websites directed to the right place and downloaded PDF files
	Testing SMS text message reminders	Ensuring reminders for medications or physician appointments were sent at the specified date and time
	Testing other logistics	Ensuring no cross-project contamination and other features (eg, nonbusiness hours response and *START* keyword to enroll)
**Concerns**		
	Messages need editing	Alerting the presence of misspellings in the SMS text message sent by the platform or suggesting editing the wording
	Need to use a different link shortener	The link shortener the team had used would be identified as spam by the cell phone carrier and blocked
	Need to embed first names in messages	The team wanted a function that automatically embedded the first name at the beginning of some SMS text messages
	Need to tailor timing to time zone	The team wondered how messages could be sent at specific times depending on participants’ time zone
	Need keyword libraries to loop	Keywords stopped sending after they reached the bottom of the library
	Need to edit *out of business hours* timing	The response on sending a message out of business hours was not set correctly
	Preference to embed links within words	The team wondered if links could be embedded within words that would open upon clicking on them

### Findings From Usability Testing (Stage 6)

Of the 5 participating caregivers in the usability testing, 4 (80%) were women. The mean age was 44.6 (SD 6.8; range 33-50) years. All participants were insured, and their mean level of education was 15.6 (SD 2.2; range 12-18) years. All 5 participants identified as Latinx, 40% (2/5) as Native American, 20% (1/5) as White, and 40% (2/5) as >1 race. All participants were born outside of the United States, including Mexico (1/5, 20%), Central America (2/5, 40%), and South America (2/5, 40%). All but one participant completed the intervention and assessments in Spanish, and their self-perceived level of spoken English was medium (2/5, 40%), high (1/5, 20%), and very high (2/5, 40%). Participants were daughters (3/5, 60%), a son (1/5, 20%), and a granddaughter (1/5, 20%) of an individual with dementia. Their average care recipient’s age was 77.0 (SD 5.1; range 72-83) years.

In general, participants completed the surveys and texting tasks without any major issues (eg, reading specific messages, using keywords, sending SMS text messages, opening links to websites and videos, and downloading PDF files). Observations of participants’ reactions during the usability testing and comments at the end of the testing revealed some minor concerns and generally positive feedback (Table 3). We addressed the concerns in various ways. First, we replaced expressions that were hard to understand (eg, 24/7 for Spanish speakers with *at any time*). Second, we added context to several SMS text messages to improve understanding. For example, we explained that a *caregiver forum* is a web platform to share experiences with other caregivers, that the content of a PDF file of a Latin American healthy recipe book alternated pages in English and Spanish, the function of specific keywords, the keyword options using simple graphics, that keywords can be sent more than once for additional messages, and that websites and other resources had a Spanish-language option. Third, we tailored the response to the keyword *STOP* (discontinuing the intervention). We also tailored the notification *CuidaTEXT* automatically sends out when a participant texts outside of business hours by including both languages within the same message because the platform did not allow separate messages in English and Spanish.

Participants shared mostly positive feedback at the end of the interview, including the following. First, satisfaction with the intervention in terms of general content, logistics, and simplicity was high. Comments included the following: “I think the program is great,” “I love the information and the testimonials,” “The messages made me feel like I’m not alone and put things into perspective,” and “The messages are simple, and the gratitude-theme messages helped.” Second, participants expressed their gratitude to the *CuidaTEXT* team for developing the intervention. Comments included the following: “Thank you for creating this type of program!” Third, they expressed their need and that of the community to use this intervention. Comments included the following: “I hope we can use it soon because we need it” and “I think it’s going to be very helpful for caregivers emotionally and personally.” The mean System Usability Scale score was 97 and ranged from 90 to 100, which is above the standard cutoff of 68. These scores indicate that the intervention’s usability holds promise.

**Table 3:** Themes, subthemes, and descriptions of topics elicited during the pilot test with 5 Latinx caregiver participants via observation or comments.

Themes and subthemes			Description
**Participants’ concerns**			
	Expressions are hard to understand	Expressions such as 24/7 or the *+* sign for *more* were hard to understand, especially for Spanish-speaking Latinx individuals	
	*CuidaTXT* is hard to read in Spanish	Spanish-speaking participants had issues pronouncing the original name *CuidaTXT* and suggested *CuidaTEXT*	
	Context is needed for understanding	Some SMS text messages required additional context or an explanation to be understood, for example, for the word *forum*	
	Branching did not work	In one instance, responding *YES* or *NO* to a specific question was not followed by a preconfigured response	
	Need to edit language of functions	The response on discontinuing the program or sending a message out of business hours was only in English	
	Need for caregiver lifestyle messages	A participant suggested adding nutrition as a healthy lifestyle action for caregivers	
**Other comments from participants**			
	Satisfaction with the intervention	Satisfaction with the intervention in general, content, logistics, and simplicity	
	Gratitude for the intervention	Expressions of gratitude for developing the intervention	
	Highlighting need for this intervention	Highlighting the need for this intervention among Latinx individuals and themselves	

### Final Product

**Figure 1:**
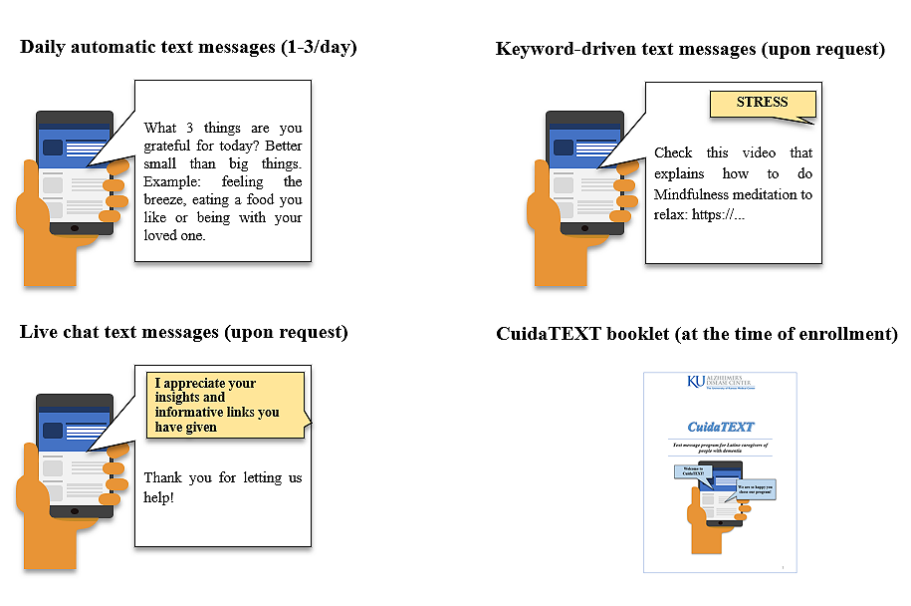
Final *CuidaTEXT* product: SMS text messaging interaction modalities and booklet.

Figure 1 summarizes the final *CuidaTEXT* product, including an example of the 3 types of SMS text messaging interaction modalities (daily automatic, keyword-driven, and live chat messages) and the reference booklet. The final version of *CuidaTEXT* includes 244 English- and 244 Spanish-language messages within the daily automatic SMS text message library. These messages will be automatically sent to all participants, starting with approximately 3 messages per day for the first 2 weeks, 2 per day for the following 2 weeks, and 1 per day for the remainder of the intervention. This daily automatic SMS text message library includes logistics messages that greet the participant on starting and completing the intervention, explain the intervention functions (eg, reminding participants of the keywords they can use for help with specific topics), and reinforce participants for being in the intervention after 2 weeks initially and monthly. The remainder of the daily automatic library includes the messages that the research team and advisory board considered the core from each domain. These domains include messages for (1) dementia education, (2) caregiver self-care messages, (3) support to and from others, (4) education about the dying and grief processes, (5) generic problem-solving strategies for behavioral symptoms, (6) specific strategies to help with the daily care of individuals with dementia, and (7) specific strategies to help address or cope with the behavioral symptoms of individuals with dementia.

*CuidaTEXT* also contains messages for 2 types of keyword-driven messages, the content keywords and menu keywords. Content keywords automatically send tips, resources, or other types of content in response to SMS text messages that include a specific keyword (eg, *STRESS* and *RESOURCES*). These keywords reflect the same domains as the daily automatic SMS text message library, except that their content is not considered core but rather an in-depth expansion for those who need further support with those domains. Menu keywords simply remind the participant which content keywords are in that category. For example, texting the menu keyword *CAREGIVER* will drive an automatic response reminding participants that content keywords within that domain include *STRESS*, *WELLBEING*, and *LIFESYLE*. Table 4 shows the function and an example of each content keyword, the menu keyword they belong to, and the number of messages in each content keyword library.

Any SMS text message other than keywords sent by participants will be received as a live chat by a bilingual and culturally proficient coach trained in dementia care. The coach will be available during business hours and will assist participants in whatever their need is (eg, additional information about a caregiver grant and programming 3-way calls with a clinic). The coach will have a bachelor’s degree or higher in a behavioral health-related area, will be trained in dementia care, and will be given a list of general contacts to find local resources (eg, Alzheimer’s Association hotline and Eldercare Locator). The final version of the *CuidaTEXT* reference booklet includes 19 pages with nine chapters: (1) What Is Dementia, (2) Signs and Symptoms of Dementia, (3) Why Focus on Latino Caregivers, (4) *CuidaTEXT* (Automatic Messages), (5) Assistant, (6) Notifications, (7) Keywords, (8) Materials, and (9) Contact Information.

**Table 4:** Keywords and their function and size of each keyword library in number of messages (n).

Content keyword (English and Spanish)	Menu keyword (English and Spanish)	Function of content keyword	Messages, n
EDUCATION and EDUCACION	None	Basic dementia information: types, stages, and impact	54
STRESS and CALMA	CAREGIVER and CUIDADOR	Strategies to cope with stress, such as relaxation	33
WELLBEING and BIENESTAR	CAREGIVER and CUIDADOR	Strategies and tips to improve well-being, such as gratitude and cognitive restructuring	30
LIFESTYLE and SALUDABLE	CAREGIVER and CUIDADOR	Tips to maintain a healthy lifestyle, such as exercising	21
FAMILY and FAMILIA	SUPPORT and APOYO	Tips to improve family communication	19
DOCTOR and MEDICO	SUPPORT and APOYO	Tips to improve communication with health providers	15
PATIENT and PACIENTE	SUPPORT and APOYO	Tips to improve communication with the individual with dementia	37
CHILDREN and NINOS	SUPPORT and APOYO	Tips to communicate with children about dementia	17
LISTENING and ESCUCHA	SUPPORT and APOYO	Strategies to improve listening skills	33
RESOURCES and RECURSOS	SUPPORT and APOYO	Contact information of resources such as support groups, legal and financial assistance, or food delivery	26
SOLVE and SOLUCION	None	General strategies to solve challenging behaviors	19
GRIEF and DUELO	None	Education on end-of-life care and tips for grieving	30
ACTIVITITES and ACTIVIDADES	CARE and CUIDADO	Tips to think of fun activities and adjust them to the abilities of individual with dementia	52
EATING and COMER	CARE and CUIDADO	Tips to make eating easier and healthier for the individual with dementia	36
DRESSING and VESTIR	CARE and CUIDADO	Tips to help the individual with dementia get dressed and groomed	18
BATHING and DUCHA	CARE and CUIDADO	Tips to help the individual with dementia take a bath or shower	24
TOILET and BANO	CARE and CUIDADO	Tips to manage the incontinence or constipation of the individual with dementia	24
MEDICATIONS and MEDICAMENTO	CARE and CUIDADO	Tips to improve medication adherence	27
HOME and CASA	CARE and CUIDADO	Tips to keep the home safe	50
DRIVE and CONDUCIR	CARE and CUIDADO	Tips to detect when it is no longer safe for the individual with dementia to drive and how to manage it	22
ANGER and ENFADO	BEHAVIOR and CONDUCTA	Tips to cope with manage the aggressive behavior of the individual with dementia	31
NERVOUS and NERVIOS	BEHAVIOR and CONDUCTA	Tips to cope with manage the anxious behavior of the individuals with dementia	33
DEPRESSION and TRISTE	BEHAVIOR and CONDUCTA	Tips to cope with manage the depressed mood of the individual with dementia	19
DELUSIONS and DELIRIOS	BEHAVIOR and CONDUCTA	Tips to cope with manage the psychotic symptoms of the individual with dementia	22
REPEAT and REPETIR	BEHAVIOR and CONDUCTA	Tips to cope with manage the repetitive behaviors of the individual with dementia	20
SLEEP and DORMIR	BEHAVIOR and CONDUCTA	Tips to improve the sleep quality of the individual with dementia	18
WANDER and DEAMBULAR	BEHAVIOR and CONDUCTA	Tips to cope with manage the wandering behavior of the individual with dementia	23
INAPPROPRIATE and INAPROPIADO	BEHAVIOR and CONDUCTA	Tips to cope with manage the inappropriate sexual behaviors of the individual with dementia	18
HOARDING and ACUMULAR	BEHAVIOR and CONDUCTA	Tips to cope with manage the hoarding behavior of the individual with dementia	12

## Discussion

### Principal Findings

This study aimed to describe the development of *CuidaTEXT*, an SMS text messaging intervention, to support Latinx family caregivers of individuals with dementia. We followed user-centered design principles to ensure the intervention’s tailoring and usability among Latinx caregivers of individuals with dementia. After a series of user-centered design stages, *CuidaTEXT*’s prototype showed a very high usability score, indicating great promise for the intervention’s feasibility and acceptability.

### Comparison With Previous Work

To our knowledge, this is the first SMS text messaging intervention for caregiver support of individuals with dementia among Latinx individuals and any other ethnic group. Very few evidence-based and culturally tailored caregiver support interventions have been developed for Latinx individuals. These interventions include fotonovelas, webnovelas, support groups, care management, and psychoeducational programs [28,45,56,57,76]. The modality of all these interventions has been individual or group face-to-face, computer-based, telephone-based, or mail-based. *CuidaTEXT* has the potential to address implementation gaps in these interventions by (1) increased accessibility compared with face-to-face or web-based interventions; (2) improved acceptability compared with phone-based interventions; (3) tailoring the content to the needs of caregivers rather than using rigid curricula; (4) addressing stigma by privately sending SMS text messages to the caregivers’ cell phone; and (5) reduced demand on the health care workforce to deliver the intervention, therefore improving fidelity and facilitating future scale-up of the intervention. Although *CuidaTEXT* was developed for Latinx individuals, similar interventions may be beneficial for other ethnic groups, especially those in rural areas, given the nearly universal cell phone ownership of most populations in the United States [31].

The advisory board suggested SMS text message content related to dementia education, social support, care, caregiver needs, community resources, and appointment reminders. These domains are most frequently included in multidomain caregiver support interventions, which have been shown to be more efficacious than single-domain interventions [1,26]. As mentioned in a recent federally commissioned report, of all interventions to improve caregiver well-being, multicomponent interventions use the most targeted components, and they possibly address at least one critical need across a wide range of individual caregiver needs, thus improving outcomes for caregiver and individuals with dementia [77].

The advisory board encouraged the inclusion of >1 family member per individual with dementia. This idea is in line with the fact that caregiving tasks and decision-making among Latinx individuals are more likely to be shared by multiple relatives of the individuals with dementia [78,79]. In fact, interventions rarely include other family members, which is likely a reflection of centering interventions on non-Latinx White caregivers [77,80]. According to our advisory board, the potential benefits of including more >1 family member may include improving caregiving quality and reducing caregiver burden.

### Limitations

This study has several limitations. First, most participants in the usability testing stage identified as the adult children of individuals with dementia, were women, were relatively highly educated, were medically insured, and had at least a medium level of English proficiency. This sample may have placed a higher focus of the refinement of the intervention on these groups than on men, spousal caregivers, and those with lower educational attainment, who lack of medical insurance, with limited English proficiency, or who may have uniquely different needs. However, most caregivers are women, and individuals from many of these other groups were represented in other stages of the development of *CuidaTEXT* (eg, advisory board). Second, the eligibility to participate in the caregiver advisory board sessions and the usability testing was based on self-report of the care recipient’s dementia status. In addition, we excluded individuals who could not read and send SMS text messages. Although the size of this group is minimal [31], future efforts could include this group by developing training to those without texting experience. Third, *CuidaTEXT* does not consider caregivers’ baseline characteristics to tailor the automated content of SMS text messages and does not tailor the timing at which SMS text messages are sent, as suggested by some advisory board participants. We consider that including keyword-driven messages addresses many of the same concerns and reduces the reliance on a baseline assessment. Any need beyond those addressed by the keyword-driven messages can also be addressed via live chat with a coach. Fourth, this study assessed the usability of the *CuidaTEXT* prototype. Although this prototype included the most relevant aspects of *CuidaTEXT*, future studies need to assess the usability of the entire intervention.

### Implications and Future Directions

This study has implications for public health, clinical practice, and research. Regarding the public health implications, *CuidaTEXT* or similar interventions have high potential for implementation, given their ubiquitous accessibility and reliance on technology rather than on human labor. Experts in dementia caregiver interventions highlight the importance of designing interventions with implementation in mind from the beginning of the intervention for its future success [81]. The user-centered design used to develop this intervention will increase the chances of this intervention being usable, acceptable, feasible, and effective in the future. Regarding clinical practice, usability testing participants described the prototype as something that was needed by them and the Latinx community. If *CuidaTEXT* proves to be effective in future studies, this intervention could be easily implemented in clinics and community organizations, by having the caregivers send an SMS text message to enroll or by having staff enter their phone numbers and names on a website. The SMS text messaging modality may be combined with other modalities to enhance its effectiveness. For example, coaches or social workers could, in addition to interacting via live chat SMS text messages, conduct ad hoc visits or calls with the caregiver. Other findings from this study might also be useful to clinicians, including the need to consider shared caregiver roles within Latinx families and other preferences. The findings reported in this manuscript may also inform future research. Future SMS text messaging studies (whether they are dementia-related or not) might decide to address the content or logistics of their interventions based on the feedback we received from the advisory board sessions or usability testing feedback. Caregiver studies might want to test the efficacy of the same caregiver support intervention to only the primary caregiver versus multiple caregivers within the same family. Future studies will test the feasibility and acceptability of *CuidaTEXT* among a diverse sample of Latinx caregivers, including variations in regional, linguistic, age, socioeconomic status, relationship to the individual with dementia, hearing functioning, and other important characteristics. This diverse representation will allow further intervention refinement, informed by qualitative analysis of SMS text messaging interactions and open-ended questions about their experiences using *CuidaTEXT*. If the future feasibility study is successful, we will conduct a fully powered randomized controlled trial to assess its efficacy.

### Conclusions

This study describes the development of *CuidaTEXT*, the first tailored SMS text messaging intervention specifically designed to support family caregivers of individuals with dementia in the Latinx community. The prototype of *CuidaTEXT* has shown very high usability, addresses Latinx caregiver needs, and has the potential for widespread implementation. The findings from several stages of the user-centered design provide useful information to guide the development and refinement of caregiver support interventions for Latinx individuals and other groups. This information contributes to efforts to address dementia disparities among Latinx individuals and gaps in the implementation of caregiver support interventions for this sizable population. We will soon test the feasibility and acceptability of this promising intervention (*CuidaTEXT*) in a 1-arm trial among Latinx family caregivers of 20 individuals with dementia (ClinicalTrials.gov NCT04316104).

## Appendix

Multimedia Appendix 1 Sample usability testing interview.
