# Supplementary material for: A Text Messaging Intervention to Support Latinx Family Caregivers of Individuals With Dementia (CuidaTEXT): Development and Usability Study
Source: JMIR Aging. 2022 Apr 28;5(2):e35625. doi: 10.2196/35625 (PMC9100381; doi:10.2196/35625)
Supplement: Multimedia Appendix 1 [file aging_v5i2e35625_app1.docx]

**Appendix 1. Sample Usability Testing Interview**

Usability Testing:

- Enrollment in text
  - Could the person enroll?
- Reading some messages and asking what they understand by them
  - Welcome to CuidaTXT, %first_name%! We're so happy you chose our program. We will text you every day to help you take care of yourself & your loved one
    - What do you understand by this message?
    - How do you like this message as introductory text?
  - We'll text you information & tips for 6 months, based on science & families' experiences. We'll refer to your relative with memory issues as Your Loved One
    - What does the “based on science and experiences” make you think?
    - What do you understand we will do when we say “we’ll refer to the person with memory issues as your loved one?”
  - The Alzheimer's Association has reliable info & support for people with memory issues. Call their free 24/7 helpline at 1-800-272-3900 or visit [link]
    - We send this message on Day 1. What would you do if you needed more information now?
    - Can you make sure the link opens on your phone?
    - Would you rather call or use the link?
- Message with link to Association forum
  - Got questions for other caregivers? Visit this forum: [link]. For in-person or remote caregiver support groups call 1-800-272-3900
    - What do you understand by this message?
    - Can you make sure the link opens on your phone?
- Use of free text
  - Can you text any question you may have to this number?
- Opening a video (ask for subtitles)
  - Hello %first_name%! Click on this link for a video with information about Alzheimer's & other dementias. It's about 20 minutes long: [link]
    - Can you open this link, please?
    - Can you find the subtitles (CC)?
- Opening a PDF
  - Have a look at this bilingual recipe book with healthy Latin American recipes: [link]
    - Can you open it?
- Keywords
  - Major keywords: EDUCATION, CARE, CAREGIVER, SOLVE, BEHAVIOR & SUPPORT. They'll display either menus of other keywords or direct tips. Try them now! %img:4245%
    - Can you see the image?
    - What would you text if you wanted more education about Alzheimer’s?
    - What would you text if you wanted to learn how to support?
  - How much information you share with children depends on their age & relationship with your loved one. For tips to discuss dementia with children, text CHILDREN
    - Can you do what this message says?
    - What did you get?
  - Most dementias end in death. People with dementia become so dependent they need to be fed or become bedbound. Text GRIEF for info to prepare for their passing
    - Can you do what this message says?
    - What did you get?
- What are the keywords you feel you’d use more?
- Message to STOP
  - Hi %first_name%! We'll send you texts daily. Some may be useful now. Others may be useful in the future. Knowledge is power! To abandon the program, text STOP
    - Did they send STOP correctly?
    - Did sending STOP discontinued the program?

[Complete survey]

- [Questions about usage of technology, relationship with care recipient, caregiver health and wellbeing, and socio-demographics]

Overall comments:

- Please provide any comments about this program:
